# SIRT3–IDH2 axis is a target of dietary fructose: implication of IDH2 as a key player in dietary carcinogen toxicity in mice colon

**DOI:** 10.1038/s12276-025-01584-0

**Published:** 2025-11-13

**Authors:** Jeong Hoon Pan, Nukhet Aykin-Burns, Kimberly J. Krager, Hyo Ri Shin, Chae Hwan Lee, Jin Hyup Lee, Byungwhi Kong, JaeEun Myoung, Kyung-Chul Choi, Jae Kyeom Kim

**Affiliations:** 1 Department of Health Behavior and Nutrition Sciences, University of Delaware, Newark, DE USA; 2 Department of Food and Nutrition, Chosun University, Gwangju, Republic of Korea; 3 Institute of Well-Aging Medicare and Chosun University G-LAMP Project Group, Chosun University, Gwangju, Republic of Korea; 4 Department of Pharmaceutical Sciences, College of Pharmacy, University of Arkansas Medical Sciences, Little Rock, AR USA; 5 Department of Food and Biotechnology, Korea University, Sejong, Republic of Korea; 6 Quality and Safety Assessment Research Unit, US Department of Agriculture, Athens, GA USA; 7 Department of Biochemistry and Molecular Biology, Brain Korea 21 Project, Asan Medical Center, University of Ulsan College of Medicine, Seoul, Republic of Korea

**Keywords:** DNA damage and repair, RNA modification

## Abstract

Recent epidemiological studies have shown that dietary fructose intake is associated with an increased risk of colorectal cancer, yet its specific molecular mechanisms in colon carcinogenesis remain underexplored. Here we investigate the molecular mechanisms by which dietary fructose contributes to colon carcinogenesis, focusing on the role of mitochondrial NADP^+^-dependent isocitrate dehydrogenase 2 (IDH2). Using an unbiased multiomics approach (transcriptomics and proteomics), liver and colon tissues from fructose-fed wild-type mice were analyzed to identify key genes involved in cancer-related pathways. In addition, human liver transcriptomic data (GSE256398) were analyzed to confirm alterations in aryl hydrocarbon receptor (AhR) signaling and the sirtuin (SIRT)3–IDH2 axis. IDH2-knockout mice were exposed to a dietary carcinogen, 2-amino-1-methyl-6-phenylimidazo(4,5-b)pyridine (PhIP), to validate IDH2’s role in colon cancer development. In vitro, fructose’s effects on SIRT3 expression and IDH2 activity were assessed. Fructose-fed wild-type mice exhibited suppressed AhR signaling, increased oxidative stress and mitochondrial dysfunction via the SIRT3–IDH2 axis. In human liver datasets, AhR-associated genes and SIRT3–IDH2 expression were reduced in metabolic dysfunction-associated steatotic liver disease and cirrhosis. The IDH2-knockout mice showed heightened DNA damage, colonic tumorigenesis and mitochondrial and glutathione-mediated detoxification disruptions following PhIP exposure. In vitro, fructose reduced SIRT3 expression and IDH2 activity, further supporting its role in promoting colon carcinogenesis. Fructose promotes colon carcinogenesis by disrupting mitochondrial function and impairing DNA damage response mechanisms, particularly through SIRT3–IDH2 axis suppression. These findings highlight the critical role of mitochondrial dysfunction in fructose-induced carcinogenesis and suggest the SIRT3–IDH2 axis as a potential therapeutic target.

## Fructose disrupts mitochondrial function in colon cancer risk

Fructose is a common sweetener, but its health effects are debated. Several studies suggest it may increase the risk of colon cancer. This study explores how fructose might contribute to cancer development. Researchers used mice to study the effects of fructose on liver and colon tissues. They focused on a gene called IDH2, which is involved in energy production and glutathione synthesis. The study found that fructose can disrupt the function of IDH2, leading to increased oxidative stress and impaired carcinogen detoxification processes. This disruption may make cells more vulnerable to DNA damage and cancer. The researchers concluded that fructose could play a role in colon cancer development by affecting mitochondrial function and detoxification pathways. Future research could explore ways to mitigate these effects, potentially offering new strategies for cancer prevention related to diet.

This summary was initially drafted using artificial intelligence, then revised and fact-checked by the author.

## Introduction

Fructose-containing sweeteners are widely used worldwide (for example, in beverages) and account for approximately 30% of total sweetener consumption in the USA^1^. Although the role of fructose in metabolic syndromes has been extensively studied, results remain conflicting, particularly in studies with food industry funding biases (for example, reviewed in ref. ^2^). Recent epidemiological evidence showed that consuming more than two servings of sweetened beverages daily has been associated with a 2.2-fold higher risk of early onset colon cancer^3^. Animal studies also support this association: a single high dose of fructose (10 g/kg body weight) increased the formation of chemical-induced colonic neoplastic lesions, such as aberrant crypt foci, compared with glucose^4^. Similarly, a recent mouse study demonstrated that a modest amount of high-fructose corn syrup promoted colon tumor growth and progression by dysregulating lipid metabolism, even in the absence of metabolic syndrome^5^. However, caution is needed when extrapolating these findings to healthy human populations because this model used adenomatous polyposis coli mutant mice that are genetically predisposed to intestinal adenoma formation, and fructose was administered after tamoxifen injection to initiate carcinogenesis^6^.

The aryl hydrocarbon receptor (AhR) is a ligand-activated transcription factor that plays a critical role in regulating xenobiotic metabolism and detoxification pathways of dietary colon carcinogens (reviewed in ref. ^7^). In brief, the activation of AhR by dietary or environmental ligands, including heterocyclic amines such as 2-amino-1-methyl-6-phenylimidazo(4,5-b)pyridine (PhIP), leads to induction of phase I and II metabolic enzymes; in the liver, these enzymes, such as cytochrome P450 isoforms, metabolize procarcinogens such as PhIP, thereby facilitating urinary excretion^8^. In that regard, the mechanisms thought to play a role in the effects of fructose in colon cancer include increased reactive oxygen species (ROS), chronic inflammation^9,10^ and the production of advanced glycation end products (which promote carcinogenesis)^11^. Furthermore, our previous work has shown that fructose suppressed hepatic AhR signaling, whereas glucose did not, indicating specific implications of fructose for carcinogen metabolism^12^. Although the above studies underscore the importance of individual dietary factors, it is unknown how red meat and fructose, which are commonly consumed together, contribute jointly to colon cancer.

In the present study, we use an unbiased multiomics approach combined with bioinformatics analyses to identify key molecular interactions involved in the colon carcinogenesis. Specifically, liver tissues from fructose-fed mice were subjected to transcriptomic and proteomic profiling to identify candidate genes that may play crucial roles in carcinogenesis. Based on these findings, the target gene—mitochondrial NADP^+^ dependent isocitrate dehydrogenase 2 (IDH2)—was deleted in mice, which were then treated with a dietary carcinogen (PhIP) to functionally validate the predictions from the omics analyses. Subsequently, colonic transcriptomics was performed to examine how IDH2 deficiency is linked to colonic carcinogenesis. Overall, this multiomics strategy enabled us to capture the complexity of these molecular interactions, providing a more holistic view of how dietary risk factors, such as fructose and PhIP, may contribute to carcinogenesis.

## Materials and methods

### Animals experiment I on ad libitum fructose-intake study

A total of 21 4-week-old male and female C57BL/6N mice (Central Lab Animal) were randomly divided into control (drinking water; four males and six females) and fructose (34% fructose water; five males and six females). In the animal experiment I, both male and female animals were included to broadly assess the effect of high fructose intake (34%) on hepatic AhR gene expression, without focusing on sex-specific differences. After acclimation, the mice were housed separately by sex under controlled temperature (23 ± 2 °C), humidity (50% ± 5%) and 12–12 h light–dark cycles in the Korea University Animal Facility (approved protocol number: KUIACUC-2018-77).

### Animals experiment II on the time-course effect of fructose on AhR

A total of 28, 4-week-old male C57BL/6N mice (Central Lab Animal) were randomly assigned to the following experimental groups: 2-week control (five males), 2-week 34% fructose (five males), 4-week control (five males) and 4-week 34% fructose (five males). In contrast to the animal experiment I, we aimed to experimentally validate this dose-dependent effect using 34% fructose for different durations and PhIP toxicity. To minimize biological variability and ensure consistency in gene expression analysis, only male animals were used in the animal experiments II through IV. After a week of acclimation to the AIN-93G diet, eight mice were euthanized at the beginning of fructose intervention as a baseline. Another 20 mice were euthanized in the second and fourth weeks. Other experimental conditions including housing, diet, anesthesia and tissue collections were identical to the experiment I.

### Animals experiments III and IV: short- and long-term PhIP-induced models

IDH2-knockout (IDH2-KO) mice and their littermate wild-type (WT) mice were maintained under the same housing conditions as described in the animal experiment I. The animal handling protocol was approved by the Institutional Animal Care and Use Committee of the University of Delaware (protocol approval number: 1354-2020-A). For a short-term study, a total of 20 mice were randomly assigned to four experimental groups: WT control group, WT + PhIP group, IDH2-KO control group and IDH2-KO + PhIP group. After acclimation, the PhIP groups received an intraperitoneal injection of PhIP (10 mg/kg of body weight, dissolved in corn oil; Toronto Research Chemicals). The injection volume of PhIP did not exceed 100 µl and was calculated on the basis of body weight. The control group mice were treated with corn oil only (10 ml/kg of body weight). At 24 h after the PhIP injection, the mice were euthanized by exposure to CO_2_ gas, followed by cardiac puncture.

For the long-term study, 60 mice were randomly divided into four groups. After the acclimation period, 100 mg/kg body weight of PhIP was orally administrated to mice in PhIP groups twice at 3-day intervals. At 4 days after the second PhIP administration, 2.5% dextran sulfate sodium (DSS)—commonly used in colorectal cancer studies for its relevance to clinical features^13,14^—was added to drinking water for 4 days, followed by 7 weeks of an observation period. Finally, all mice were euthanized by exposure to CO_2_ gas. Daily food intake was measured, and behavioral activity was monitored to assess animal health and well-being after PhIP or PhIP+DSS treatment. The collected tissues were stored at −80 °C in the RNALater solution or fixed with 10% neutral buffered formalin solution for further analyses.

### Cell culture validation

Mouse hepatocyte (AML12; American Type Culture Collection) was cultured in Dulbecco’s modified Eagle medium/F12 media supplemented with 10% fetal bovine serum, 40 ng/ml dexamethasone and insulin–transferrin–selenium-G supplement (Invitrogen). The cells were incubated at 37 °C with 5% CO_2_ and water saturation. The cells were treated with 5 mM fructose for 24 h, and then, the cells were collected for further analyses. In addition, to validate the role of fructose-induced ROS on AhR suppression, *N*-acetylcysteine (5 mM for 1 h) was treated to the cells followed by hydrogen peroxide (0.5 mM for 8 h), and the cells were collected for the measurement of AhR-related gene expression.

### RNA extraction and mRNA expression analysis

The tissue or cellular RNAs were isolated using the RNeasy Plus Universal Mini Kit (Qiagen) as described^12^. For 84 messenger RNAs related to DNA damage and repair, RT^2^ Profiler PCR Array (Qiagen) was used.

### Transcriptomics

The total RNA isolated from liver and colon tissues was used for RNA sequencing. The transcriptomic analysis was performed on hepatic samples (*n* = 5 per group; two males and three females) and colonic samples (*n* = 6 per group; three males and three females) using a 1 × 50 bp single-end read on an Illumina HiSeq system (Illumina), as described previously^15^. The total mapped counts were log_2_-transformed on the basis of the reads per million to stabilize the variance. The normalized values were then further processed to identify differentially expressed genes (DEGs).

### Secondary analysis of human liver transcriptome datasets

The potential disruption of AhR signaling and the sirtuin (SIRT)3–IDH2 axis was investigated in the context of xenobiotic detoxification. Whereas our animal and cellular experiments focus on fructose-induced effects, we sought to determine whether similar molecular alterations occur in human liver disease by analyzing publicly available transcriptomic data. To support our hypothesis that fructose impairs AhR-mediated detoxification and suppresses the SIRT3–IDH2 axis, we first conducted a secondary analysis of human liver transcriptomic data (GSE256398, https://www.ncbi.nlm.nih.gov/geo/query/acc.cgi?acc=GSE256398). This analysis aims to contextualize our experimental results within human pathology, highlighting the potential implications of fructose-induced metabolic dysfunction in disease progression. In addition, this human data will serve as the foundation for further validation in our animal and cellular experiments.

### Proteomics

Protein fractions were isolated from collected liver tissues obtained from animal experiment I. The isolated proteins were reduced, alkylated and digested using a filter-aided sample preparation, as previously described^16^. The eluted peptides were ionized via electrospray (2.15 kV) and subjected to mass spectrometric analysis using an Orbitrap Fusion Tribrid mass spectrometer (Thermo Fisher Scientific) with multinotch MS^3^ parameters^17^. The data were acquired in top-speed profile mode with a resolution of 240,000, covering a range of 375 to 1,500 *m*/*z*. Following a collision-induced dissociation, the data were acquired using the ion trap analyzer in centroid mode, ranging from 400–2,000 *m*/*z*. Up to ten tandem mass spectrometry precursors were selected for higher energy collision dissociation (normalized collision energy of 65.0), followed by MS^3^ reporter ion data acquisition in profile mode with a resolution of 30,000 over a range of 100–500 *m*/*z*.

The proteins were identified, and the reporter ions were quantified using MaxQuant software (Max Planck Institute) with a parent ion tolerance of 3 ppm, a fragment ion tolerance of 0.5 Da and a reporter ion tolerance of 0.03 Da. Protein identifications were accepted with a false discovery rate below 1% and at least two identified peptides. The results were compiled using the Scaffold program (Proteome Software). The proteins with a *P* value <0.05 and a fold change >1.5 were considered significantly different, resulting in 179 differentially expressed proteins, which were further analyzed using Ingenuity Pathway Analysis (IPA; Qiagen).

### Western blot

The protein expression was analyzed using western blotting, as we described elsewhere^18^.

### Immunoprecipitation of IDH2

Immunoprecipitation for acetylated IDH2 was carried out using magnetic beads. Crude protein extracts from hepatocytes treated with either distilled water or 5 mM fructose were incubated with anti-acetyl-IDH2 antibody bound to the magnetic beads. Eluted antigens were utilized for western blot analysis.

### Pathophysiological analyses

Fixed and dehydrated colon tissues were cut open and laid flat in between two cover slides. The slide sandwiches were frozen on dry ice. For embedding, Tissue-Tek Optimal Cutting Temperature (OCT) compound (Sakura Finetek) was added to a plastic mold to make an OCT block with a flat surface. Colon tissues in the slide sandwich were transferred to the flat side of the OCT block with the mucosal surface facing up. The mucosal surface was covered with a generous amount of OCT compound. The embedded colon tissues were cut into 5-μm sections and stained with hematoxylin and eosin staining for morphological observation. The stained tissue sections were examined using a 5× objective on a Leica DM 500 microscope equipped with a Leica ICC50E (Leica Camera).

For immunofluorescence analyses, en face colon sections embedded in OCT blocks were incubated with specific primary antibodies overnight at 4 °C, followed by washing cycles with phosphate buffered saline. Subsequently, tissue sections were incubated with fluorophores-conjugated secondary antibody for one hour at room temperature. The tissue sections were counterstained with 4′,6-diamidino-2-phenylindole and sealed with cover glass. The obtained images were analyzed using a confocal microscope (Carl Zeiss AG).

### Enzyme activity assays

For IDH2 activity, the mitochondria of hepatocytes were isolated using a mitochondrial isolation kit for cultured cells (Thermo Scientific) according to instructions of the manufacturer. IDH2 activity was measured using the IDH Activity Assay Kit (Sigma-Aldrich) according to instructions of the manufacturer. The Glutathione Reductase Assay kit (Sigma-Aldrich) was also used to measure the hepatic glutathione (GSH) reductase activity in response to fructose treatment.

### GSSG/GSH and NADPH/NADP assays

The ratios for oxidized GSH (GSSG)/GSH and NADPH/NADP^+^ were measured using the GSH/GSSG ratio detection assay kit and NADP^+^/NADPH assay kit (Abcam).

### Bioinformatics and statistical analyses

As for secondary analyses to compare the dose-dependent effects of fructose intake on hepatic carcinogen metabolism, NCBI Gene Expression Omnibus DataSets were utilized to retrieve related transcriptomics datasets. GSE92502 and GSE51885 met our search criteria (that is, fructose-fed C57BL/6 mice, lower or higher fructose dose than our study); thus, DEGs were acquired. Each DEG was subjected to the IPA analysis, followed by a comparison analysis of the three datasets.

Using the R software, partial least squares discriminant analysis (PLS-DA) was performed on the colonic transcriptomics dataset before DEG analysis. Our DEGs and differentially expressed proteins were then analyzed using multiple bioinformatics tools as follows. First, the gene ontology analysis was performed using the DAVID tool. Specifically, DEGs from the hepatic transcriptome were analyzed with the DAVID tool to identify enriched terms for the Kyoto Encyclopedia of Genes and Genomes (KEGG) pathways. Moreover, the DEGs were subjected to analysis using the IPA software (Qiagen), where the core analysis feature of the software was performed to predict related pathways, including canonical pathway analysis and upstream regulator analysis.

For all other markers, data are presented as mean ± s.e.m. We assessed whether the data followed a normal distribution using the Shapiro–Wilk test. When the data did not fit a normal distribution, the Mann–Whitney test was applied. Comparisons between two groups were analyzed using an unpaired *t*-test, whereas data involving two independent variables and one dependent variable were first analyzed using a two-way ANOVA followed by Tukey’s multiple comparison. In cases where a significant interaction effect was not observed, a one-way ANOVA was conducted, followed by Fisher’s least significant difference test. A *P* value of 0.05 or less was considered statistically significant (GraphPad Software).

## Results

### Fructose-associated suppression of AhR signaling and SIRT3–IDH2 axis in human liver disease

**Fig. 1:**
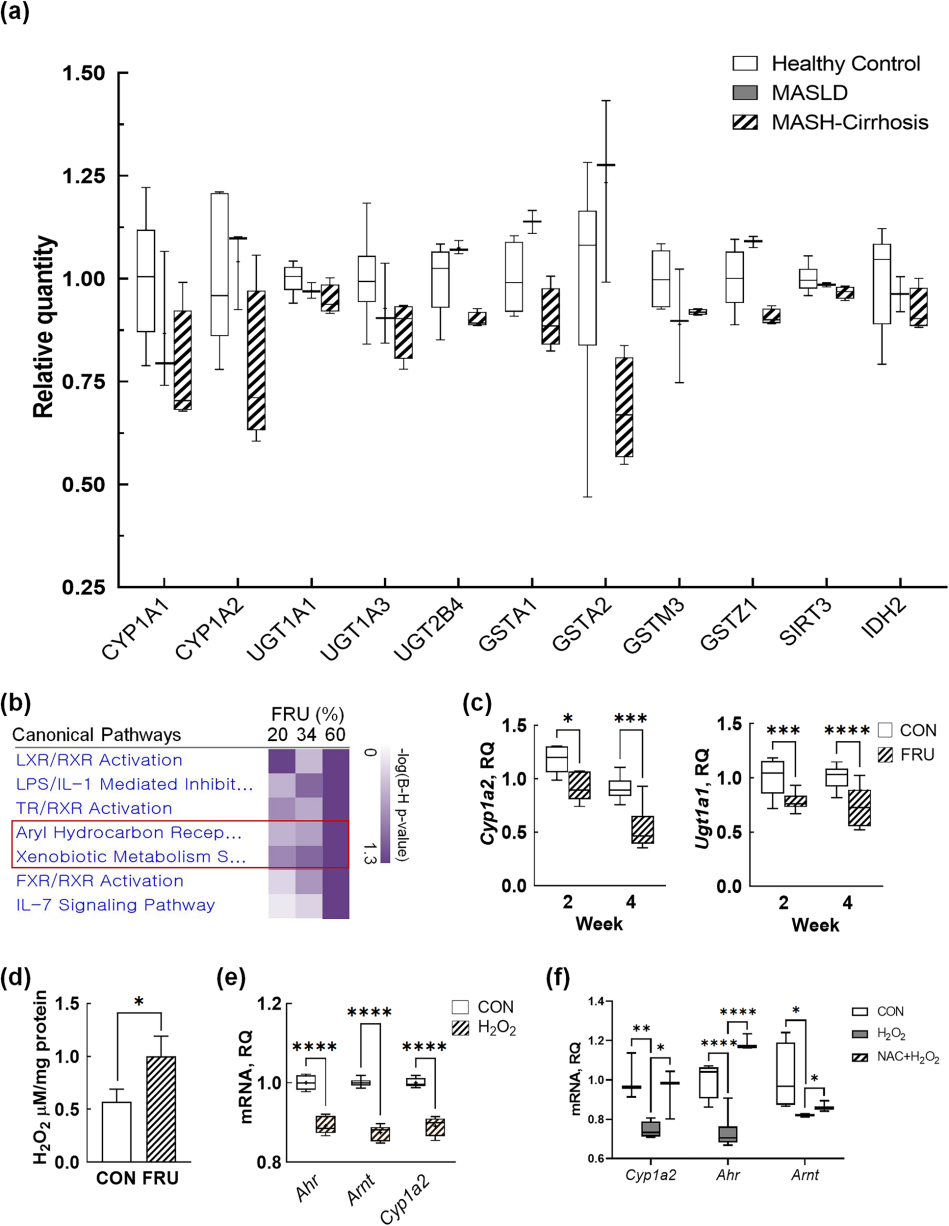
Fructose suppresses hepatic AhR signaling pathway. **a** The changes in AhR-associated genes, phase I/II detoxification enzymes, SIRT3 and IDH2 expression in the liver transcriptome analysis of patients with MASLD and cirrhosis. **b** A comparison analysis of three hepatic transcriptome datasets with different fructose doses. **c** The time course effect of fructose on *Cyp1a2* and *Ugt1a1* mRNA expression in the liver tissues of mice fed 34% fructose water for 2 and 4 weeks. **d**–**f** In vitro validation using alpha mouse liver (AML)12 hepatocytes: **d** the ROS-inducing effect of fructose, **e** ROS-mediated suppression of AhR signaling, and (**f**) reversal of ROS-mediated AhR suppression by antioxidation *N*-acetylcysteine (NAC). The data are presented as mean ± s.e.m. (*n* = 6 per group). A *P* value of 0.05 or less was considered statistically significant; *P < 0.05, ***P < 0.001, ****P < 0.0001. CON control, FRU fructose, MASH-cirrhosis metabolic dysfunction-associated steatohepatitis cirrhosis, RQ relative quantity. AhR aryl hydrocarbon receptor, AML alpha mouse liver, CON control, FRU fructose, IDH2 isocitrate dehydrogenase 2, MASH-cirrhosis metabolic dysfunction- associated steatohepatitis cirrhosis, MASLD metabolic dysfunction-associated steatotic liver disease, ROS reactive oxygen species, RQ relative quantity, SIRT3 sirtuin 3.

In our analysis of human liver transcriptomic data, we observed a marked downregulation of AhR-associated genes in patients with metabolic dysfunction-associated steatotic liver disease (MASLD) and cirrhosis compared with healthy controls, particularly those involved in xenobiotic metabolism, including phase I (CYP1A1 and CYP1A2) and phase II (UGT1A1, UGT1A3, UGT2B4, GSTA1, GSTA2, GSTM3 and GSTZ1) detoxification enzymes (Fig. 1a). The suppression suggests a progressive decline in hepatic detoxification capacity, potentially increasing the susceptibility to carcinogen accumulation. In addition, we observed a trend of decrease in SIRT3 (*P* = 0.06) and IDH2 (*P* = 0.05) expression in MASLD and fibrosis, with the most pronounced reduction in patients with cirrhosis.

### Fructose suppresses the xenobiotic AhR signaling pathway via ROS production

Previously, we showed that 34% fructose intake suppresses the AhR signaling pathway in the liver^12^. The AhR signaling pathway is a key pathway that governs genes (for example, CYPs) related to carcinogen metabolism in the liver^19^. Interestingly, we found that the chemical carcinogenesis–DNA adduct KEGG pathway is predicted enriched in the fructose group, which includes PhIP-DNA adduct-mediated colon cancer (Supplementary Fig. 1). The prediction is probably due to changes in CYPs by fructose intake as highlighted as key genes of the pathway. As we reported, a few key genes governed by the AhR signaling pathway were downregulated including CYPs^12^.

To further explore a dose-dependent implications of fructose intake on hepatic carcinogen metabolism, our transcriptomics dataset (34% fructose) was compared with two different publicly available RNA sequencing datasets that were treated with lower (20%; GSE92502) or higher (60%; GSE51885) fructose levels than our condition. In this secondary analysis, the AhR signaling pathway and xenobiotic metabolism signaling pathway were dose-dependently enriched (Fig. 1b). In a follow-up validation study, we noted that *Cyp1a2* and *Ugt1a1* mRNA expressions were decreased in fructose-fed mice liver tissues; there was a stronger statistical significance when mice were fed fructose for longer periods (Fig. 1c). Given that fructose is a known ROS-inducer and an interaction between ROS and AhR is well established, the suppression of AhR genes is probably related to fructose-induced oxidative stress under our conditions. Confirming the previous studies as well as our speculation, the fructose group presented a significantly increased hydrogen peroxide level (Fig. 1d); furthermore, mRNAs for AhR signaling-related genes (that is, *Ahr*, *Arnt* and *Cyp1a2*) were decreased in response to oxidative stress induced by hydrogen peroxide in AML12 hepatocytes (Fig. 1e). Lastly, to further elucidate the causal relationship between ROS production and suppression of the AhR signaling pathway, cells were treated with the antioxidant *N*-acetylcysteine, which successfully rescued the expression of AhR signaling-related genes (Fig. 1f).

### Fructose-induced mitochondrial dysfunction is associated with SIRT3–IDH2 axis

**Fig. 2:**
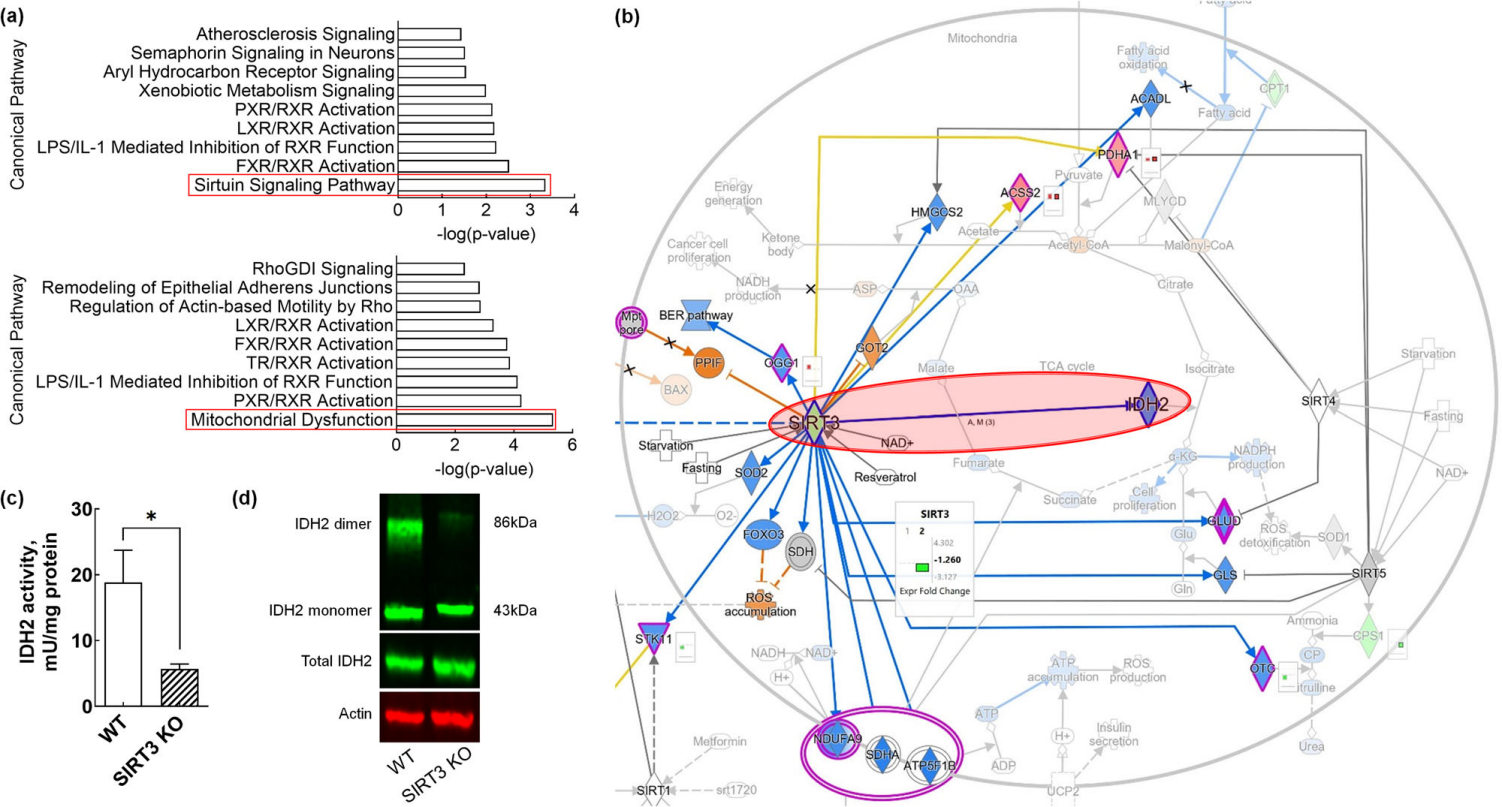
Fructose inhibits activities of both mitochondrial SIRT3 and IDH2. **a** Top canonical pathways enriched in the hepatic transcriptome (upper) and proteome (lower) of fructose-fed mice. **b** Ingenuity pathway analysis (IPA) map showing sirtuin 3 (SIRT3)-centered regulatory network in the mitochondrial signaling context. **c** Measurement of isocitrate dehydrogenase 2 (IDH2) enzymatic activity in hepatocytes from wild-type (WT) and SIRT3 knockout (KO) mice. **d** Western blot images showing IDH2 dimer (~86 kDa) and monomer (~43 kDa) in WT and SIRT3 KO hepatocytes. The data are presented as mean ± s.e.m. (*n* = 6 per group). A *P* value of 0.05 or less was considered statistically significant; *P < 0.05. IDH2 isocitrate dehydrogenase 2, KO knockout, SIRT3 sirtuin 3, WT wild type.

In our hepatic transcriptomics and proteomics datasets, SIRT signaling pathway and mitochondrial dysfunction were predicted the most enriched canonical signaling pathways in fructose-fed mice, respectively (Fig. 2a). SIRTs are a family of proteins that include several subforms, such as SIRT1, SIRT3 and SIRT5, each of which plays distinct roles in cellular processes. Among these, SIRT3 is primarily localized in the mitochondria and is well known to regulate mitochondrial function by deacetylating enzymes involved in energy metabolism, such as IDH2. The SIRT3 plays a critical role in maintaining mitochondrial integrity and protecting against oxidative stress, particularly by regulating mitochondrial dynamics, metabolic syndromes and ROS production.

Interestingly, according to our transcriptomics dataset, mitochondrial SIRT3 was predicted decreased in FRU-fed mice, leading to a suppression of IDH2 (Fig. 2b). As aforementioned, the SIRT3 is a mitochondrial NAD^+^-dependent deacetylase that directly deacetylates IDH2, which enhances the enzymatic activity of IDH2, thereby increasing its capacity to produce NADPH^20^. Therefore, we explored changes in IDH2 activity in response to SIRT3 KO to elaborate the mitochondrial SIRT3–IDH2 axis. As hypothesized, the IDH2 activity and dimerization of IDH2 were significantly decreased in SIRT3-KO mice, showing a direct regulatory mechanism of IDH2 (Fig. 2c, d).

#### IDH2-deficient mice are more sensitive to DNA damage response from short-term and long-term exposures to a dietary carcinogen

Once we confirmed the implications of fructose intake on IDH2 (via SIRT3), we further aimed to validate the predicted the KEGG pathway, chemical carcinogenesis–DNA adduct. Of the four aromatic amines and amides included in the KEGG pathway (namely, 4-aminobiphenyl, PhIP, 2-amino-3-methylimidazo(4,5-f)quinoline (IQ) in Supplementary Fig. 1 and 2-amino-3,8-dimethylimidazo(4,5-f)quinoxaline (MelQx) in Supplementary Fig. 1), involved in the pathway, we chose PhIP-induced colon cancer model for the validation as it is the most mass abundant carcinogen present in well-done meats^21^. PhIP can be detoxified via GSTs-mediated conjugation of GSH in the liver; hence, GSH is known to be protective against the production of DNA adduct and possibly colon carcinogenesis^22^. As expected, the depletion of hepatic GSH is a hallmark of IDH2 deficiency^23^, which led us to explore an implication of IDH2 in PhIP toxicity.

**Fig. 3:**
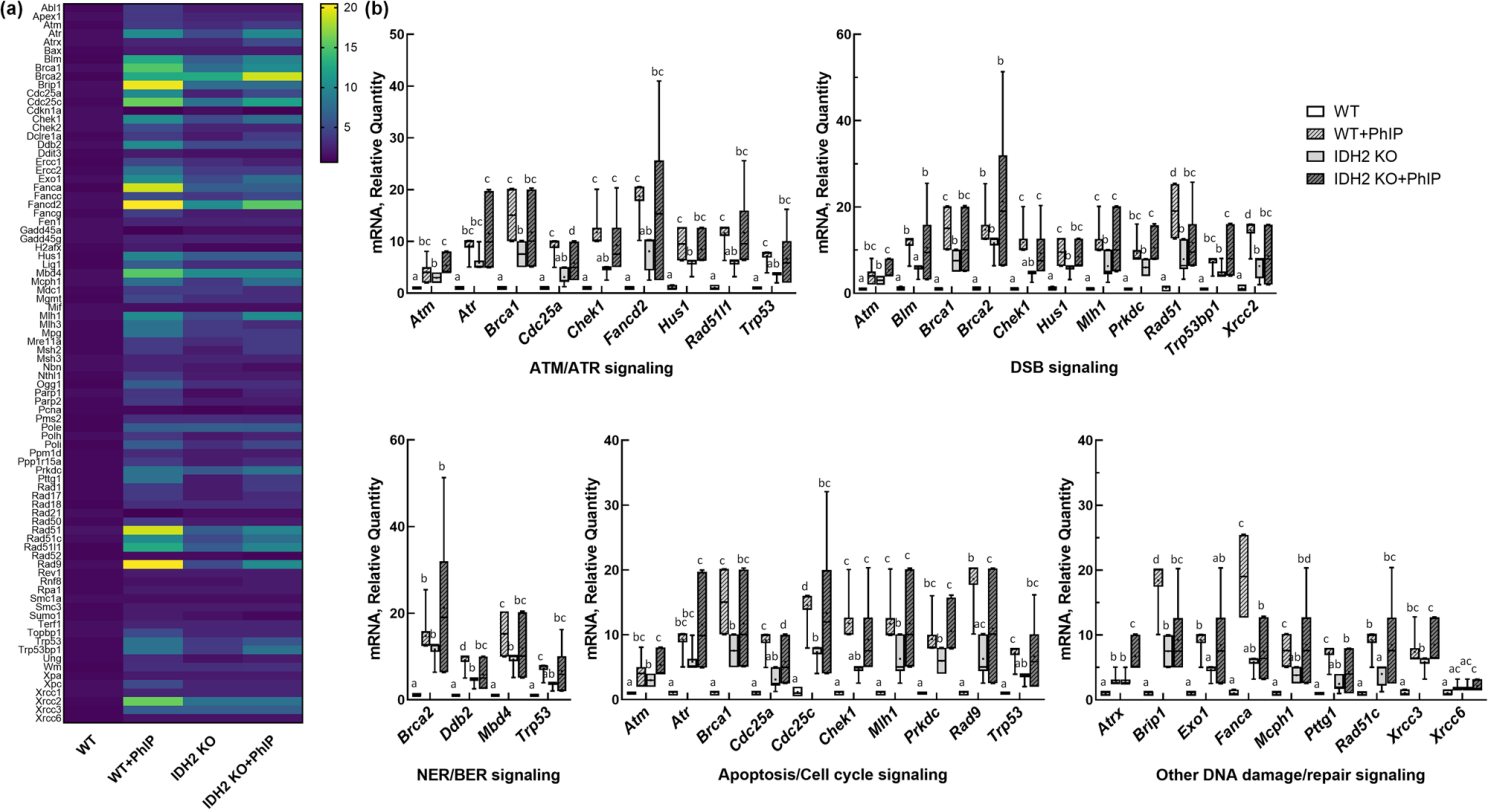
IDH2 KO exacerbates expressions of colonic DNA damage and repair genes in response to short-term PhIP exposure. **a** A heat map analysis of 86 genes associated with DNA damage and repair signaling in WT, WT + PhIP, IDH2-KO and IDH2-KO + PhIP groups. **b** The mRNA expressions for categorized molecules involved in different DNA damage and repair signaling; ataxia telangiectasia mutated (ATM)/ATR signaling, double-strand breaks (DSB) signaling, nucleotide excision repair (NER)/base excision repair (BER) signaling, apoptosis/cell cycle signaling and others. The data are presented as mean ± s.e.m. The different letters indicate a statistical difference between groups (*n* = 6 per group). A *P* value of 0.05 or less was considered statistically significant. ATM Ataxia telangiectasia mutated, ATR Ataxia telangiectasia and Rad3 related, BER base excision repair, DSB double strand break, KO knockout, IDH2 isocitrate dehydrogenase 2, NER nucleotide excision repair, PhIP 2-amino-1-methyl-6- phenylimidazo(4,5-b)pyridine, WT wild type.

In this study, we comprehensively assessed 86 genes involved in DNA damage signaling pathways in both WT and IDH2-KO mice following a 24-h exposure to PhIP. A heat map highlighted several key genes related to DNA damage signaling that were notably impacted by PhIP treatment in the colon of WT mice (Fig. 3a). Moreover, Fig. 3b shows 28 genes with statistically significant differences across the experimental groups (Fig. 3b). IDH2 deficiency in the colon led to the upregulation of several mRNAs, although the effects were not as pronounced as those induced by PhIP exposure alone. Interestingly, in the IDH2-KO mice, several mRNA levels (for example, *Atr*, *Atrx* and *Brca2*) were further elevated following PhIP treatment compared with the WT + PhIP group. However, the majority of mRNA levels tended to decrease in the IDH2-KO + PhIP group relative to the WT + PhIP group. These findings suggest potential interactions between PhIP exposure and IDH2 deficiency, with complex effects on DNA damage signaling pathways.

**Fig. 4:**
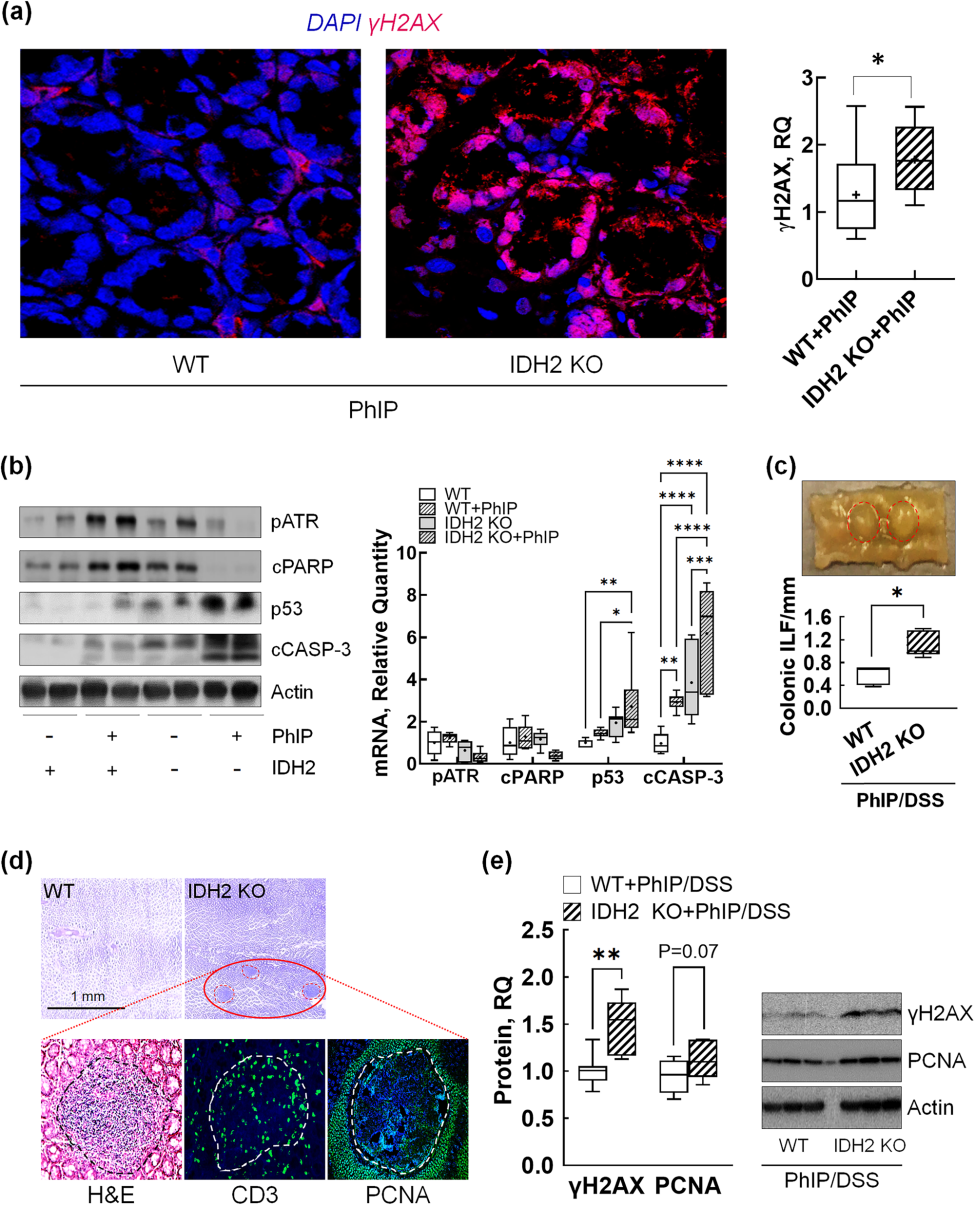
IDH2 KO promotes colonic DNA damage and ILF formation in response to short- and long-term PhIP exposure. **a** The representative images and quantification of γH2AX immunofluorescence in colon tissue sections from mice short-term exposed to PhIP. **b** The representative bands and quantification of DNA damage-related protein expressions in colon tissues from mice short-term exposed to PhIP. **c** A macroscopic image of colonic ILF and their quantification and identification of the ILF (**d**) using hematoxylin and eosin (H&E) staining and immunofluorescence of cluster of differentiation 3 (CD3) and proliferating cell nuclear antigen (PCNA) from mice long-term exposed to PhIP/DSS. **e** The representative bands and quantification for γH2AX and PCNA protein expressions in colon tissues from mice exposed to long-term PhIP/DSS. The data are presented as mean ± s.e.m. The asterisk indicates statistical differences between groups (*n* = 10 per group). A *P* value of 0.05 or less was considered statistically significant. RQ relative quantity. CD3 cluster of differentiation 3, cCASP-3 cleaved caspase 3, cPARP cleaved poly(ADP-ribose) polymerase, DSS dextran sulfate sodium, γH2AX gamma phosphorylated histone H2AX, H&E hematoxylin & eosin, IDH2 isocitrate dehydrogenase 2, ILF isolated lymphoid follicles, KO knockout, pATR phosphorylated Ataxia Telangiectasia and Rad3 related protein, PCNA proliferating cell nuclear antigen, PhIP 2- amino-1-methyl-6-phenylimidazo(4,5-b)pyridine, p53 tumor protein p53, RQ relative quantity, WT wild type.

Next, it was further demonstrated that IDH2-KO mice exhibited significantly higher levels of colonic gamma phosphorylated histone H2AX (γH2AX), tumor protein p53 (p53) and cleaved caspase 3 (cCASP-3) compared with their WT littermates 24 h after PhIP exposure (Fig. 4a, b). Notably, whereas PhIP treatment activated phosphorylated ataxia telangiectasia and Rad3-related protein (pATR) and cleaved poly(ADP-ribose) polymerase (cPARP) in WT mice, PhIP exposure in the IDH2-KO mice led to the suppression of these proteins. To confirm the implications of IDH2 deficiency in PhIP-induced toxicity from the short-term PhIP-induced model, we examined the effects of long-term PhIP exposure (8 weeks) in IDH2-KO mice; the mice were killed 7 weeks after receiving a 1-week PhIP/DSS treatment. Interestingly, the IDH2-KO mice developed more colonic isolated lymphoid follicles (ILFs), as identified by CD3 expression (Fig. 4c, d). Furthermore, higher expression levels of γH2AX and PCNA proteins were observed in the IDH2-KO mice compared with WT littermates (Fig. 4e), aligning with the results of the short-term PhIP exposure study.

### IDH2 KO exacerbates PhIP-induced colonic damages via multiple networks

**Fig. 5:**
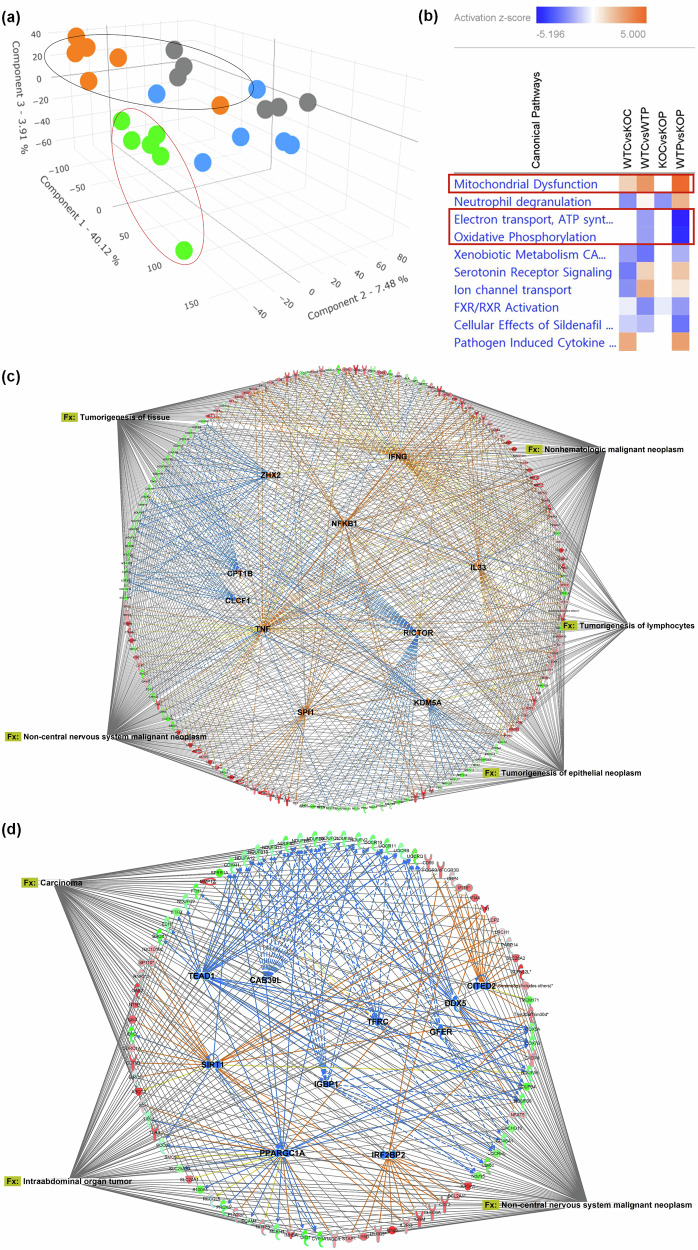
Colonic transcriptomics reveals that IDH2 KO affects several organismal injury and abnormalities related diseases and biological functions. **a** A PLS-DA plot for the WT control (WTC; gray color), WT + PhIP (WTP; blue color), IDH2-KO control (KOC; orange color) and IDH2-KO + PhIP (KOP; green color) groups long-term exposed to PhIP/DSS. **b** A comparison analysis of four groups, WTC, WTP, KOC and KOP for top ten canonical pathways of colonic transcriptome. The top ten predicted activated (**c**) and top ten predicted inhibited upstream regulators (**d**) and their association networks with diseases and biological functions. DSS dextran sulfate sodium, IDH2 isocitrate dehydrogenase 2, KO knockout, KOC IDH2 knockout control, KOP IDH2 knockout + PhIP, PhIP 2-amino-1- methyl-6-phenylimidazo(4,5-b)pyridine, PLS-DA partial least squares discriminant analysis, WT wild type, WTC wild type control, WTP wild type + PhIP.

Our findings clearly indicate that IDH2 KO exacerbates PhIP-induced colonic DNA damage and possibly promotes colon carcinogenesis. However, the deletion of a certain gene results in multifaceted effects in general; thus, colonic transcriptomics of the WT, WT + PhIP, IDH2-KO and IDH2-KO + PhIP groups was carried out to reveal the mechanistic potentials of IDH2 KO. The transcriptomics dataset was applied to PLS-DA, a supervised classification method extending the partial least squares algorithm to determine axes, which explains the most variance in both predictors and the response variables (Fig. 5a). As shown, the IDH2-KO + PhIP (Fig. 5, green) group seems well separated and well clustered in the tilted three-dimensional plot, particularly from the IDH2-KO group (Fig. 5, orange).

Next, the DEGs from the colonic transcriptomics dataset were utilized for pathway prediction analyses using the IPA. Based on a comparison analysis of the four groups, the mitochondrial function-related pathways are important to note (Fig. 5b). Both IDH2 deletion and PhIP affect mitochondrial dysfunction, leading to an exacerbated dysfunction of mitochondria in the IDH2-KO + PhIP group. Here, the complex content is systematically deconstructed and explained in each comparison for clarity and understanding. When WT and IDH2-KO groups were compared (see red and green colors, as they are actual increased or decreased genes in DEGs, respectively, and orange and blue colors are predictions (Supplementary Fig. 4)), IDH2-KO-mediated mitochondrial dysfunction might be more focused on complex IV and V (Supplementary Fig. 2), but PhIP is likely to affect complex I and IV seen in the comparison between WT and WT + PhIP (Supplementary Fig. 3). These effects may lead to the overall suppression of the electron transport chain when IDH2-KO and PhIP are applied together (Supplementary Fig. 4). Interestingly, PhIP treatment in IDH2-KO mice appeared not to be effective on mitochondrial dysfunction compared with IDH2-KO mice (Fig. 5b). This cannot be clearly addressed in the present study. However, it is possible that PhIP might have triggered a compensatory response aimed at maintaining cellular energy and survival under stress conditions resulting from PhIP treatment. In fact, our data somehow support the hypothesis. Specifically, a reason for that activation status of mitochondrial dysfunction in the comparison between IDH2 KO and IDH2 KO + PhIP was due to that recovery of multiple genes in the complexes, which resulted in less significance (Supplementary Fig. 5). Other mitochondrial pathways (that is, electron transport, ATP synthesis and heat production by uncoupling proteins and oxidative phosphorylation) were not significantly affected by IDH2 KO, but they were aggravated when PhIP was treated in IDH2-KO mice. We speculate that the mitochondrial dysfunction caused by IDH2 KO might be from other signaling pathways. In fact, several canonical pathways altered by IDH2 KO were related to mitochondrial dysfunction, although they were not highlighted. For instance, the tricarboxylic acid (TCA) cycle and respiratory electron transport and glucose metabolism were predicted inhibited in IDH2-KO mice, which are crucial pathways for ATP production (Supplementary Table 1). A previous report supports the speculation, as IDH2 deficiency led to the depletion of ATP in different cells^24,25^.

**Fig. 6:**
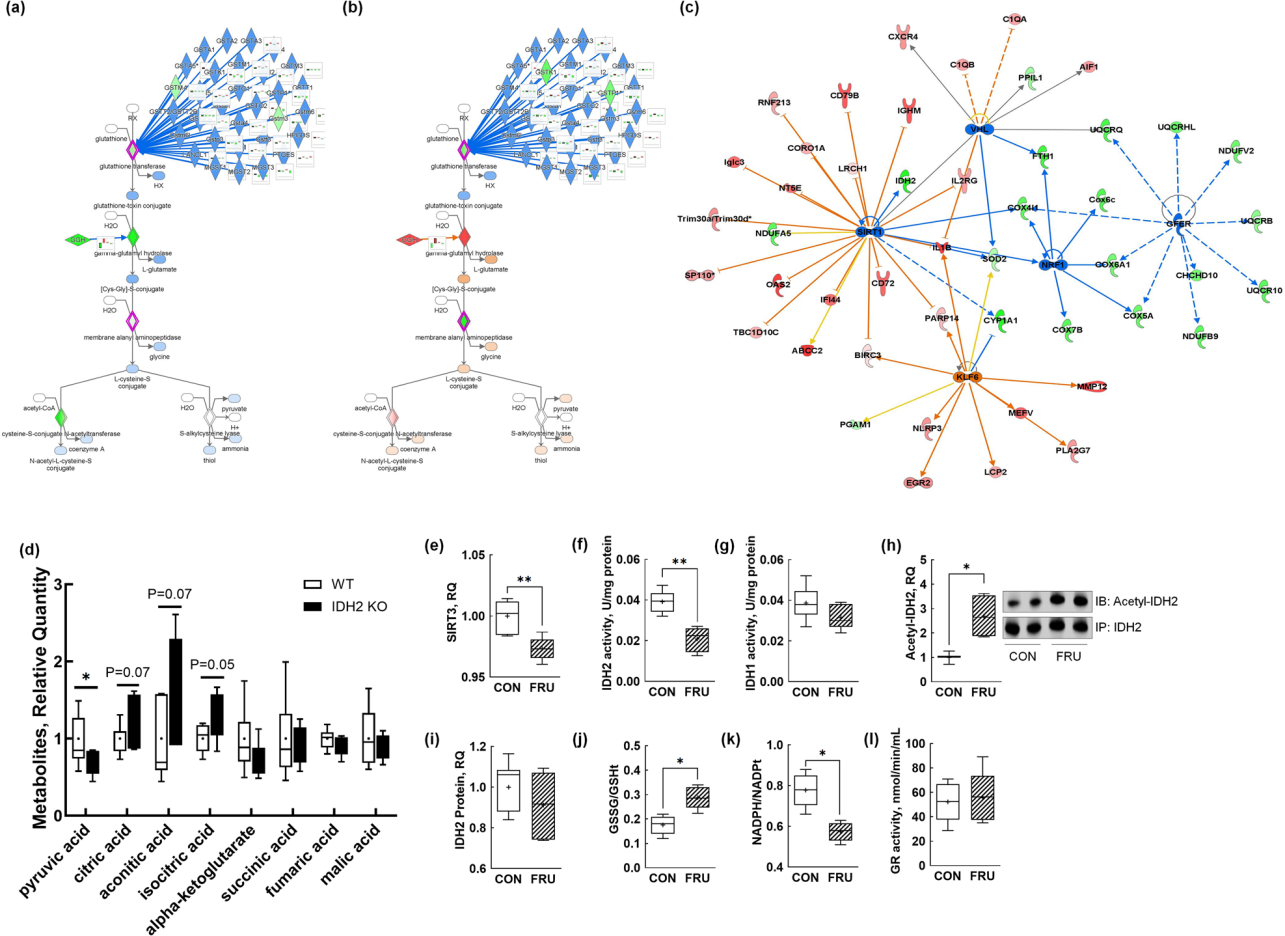
IDH2 deletion and fructose-mediated suppression of the SIRT3–IDH2 axis disrupt GSH-mediated detoxification and redox homeostasis. **a** The networks for the GSH-mediated detoxification pathway in WT colon tissues. **b** The networks for the GSH-mediated detoxification pathway in IDH2-KO colon tissues. **c** A merged network of upstream regulators linked to the GSH-mediated detoxification pathway. **d** The key TCA cycle metabolites in the plasma of mice to validate the IDH2-KO-mediated metabolic shift. **e** The suppression of SIRT3 protein expression in response to the fructose treatment in hepatocytes. **f**, **g** The inhibition of mitochondrial IDH2 activity (**f**) with unchanged cytosolic IDH1 activity (**g**) by fructose treatment in hepatocytes. **h** The increase in acetylation of IDH2 in response to fructose treatment in hepatocytes. **i** The unchanged IDH2 protein expression by the fructose treatment in hepatocytes. **j** The GSH depletion induced by fructose, measured by the ratio of GSSG to GSH. **k** The NADPH depletion induced by fructose, measured by the ratio of NADPH to NADP⁺. **l** The unchanged GR activity by fructose treatment in hepatocytes. The data are presented as mean ± s.e.m. (*n* = 3–6 per group). A *P* value of 0.05 or less was considered statistically significant; *P < 0.05, **P < 0.01. GR GSH reductase. GSH glutathione, GSSG oxidized glutathione, GR glutathione reductase, IDH1 isocitrate dehydrogenase 1, IDH2 isocitrate dehydrogenase 2, KO knockout, NADP⁺ oxidized nicotinamide adenine dinucleotide phosphate, NADPH nicotinamide adenine dinucleotide phosphate, PhIP 2-amino-1-methyl-6-phenylimidazo(4,5-b)pyridine, SIRT3 sirtuin 3, TCA tricarboxylic acid, WT wild type.

As we mentioned, GSH plays a crucial role in PhIP detoxification, and IDH2 is highly associated with GSH recycling via producing NADPH^26^. Importantly, GSH-mediated detoxification pathway was predicted to be inhibited by IDH2 KO (Fig. 6a), whereas PhIP activated it (Fig. 6b). Moreover, several upstream regulator molecules were associated with the pathway (Fig. 6c). The inhibition of GSH in the IDH2-KO group was reasonable to expect, given that the KO of IDH2 induces the reductive TCA cycle^27^. Our study also confirmed that IDH2 deletion resulted in a shift of the oxidative TCA cycle to reductive as evidenced by higher plasma levels of citrate, aconitate and isocitrate and a lower level of α-ketoglutarate in IDH2-KO mice (Fig. 6d). However, GSH-mediated detoxification mainly occurs in the liver tissue, and the suppression of hepatic GSH is a well-known event in response to fructose intake^28,29^. Furthermore, we speculated that fructose-induced GSH depletion could be due to the suppression of the SIRT3–IDH2 axis, leading us to execute validation in vitro studies using hepatocytes.

As expected, the fructose treatment led to a reduction in SIRT3 protein expression (Fig. 6e) and a corresponding decrease in IDH2 enzyme activity in mouse hepatocytes (Fig. 6f), whereas the isocitrate dehydrogenase 1 (IDH1) activity remained unaffected (Fig. 6g). This indicates that fructose specifically impacts the SIRT3–IDH2 axis. Supporting this, the fructose treatment increased the acetylation of IDH2 (Fig. 6h) despite no changes in IDH2 protein levels (Fig. 6i), further confirming that fructose acts through SIRT3-mediated deacetylation rather than altering IDH2 expression per se. In addition, the fructose treatment reduced intracellular levels of NADPH and GSH (Fig. 6j, k), key molecules involved in antioxidant defense, yet had minimal impact on NADPH-consuming GSH reductase activity (Fig. 6l). This suggests that the depletion of GSH is more likely due to impaired NADPH production via the SIRT3–IDH2 axis rather than increased consumption. Taken together, these findings strongly indicate that fructose-induced hepatic GSH depletion is closely linked to the inhibition of the SIRT3–IDH2 pathway.

## Discussion

AhR is activated by binding to ligands such as environmental pollutants including polycyclic aromatic hydrocarbons^30^. Upon ligand binding, AhR translocates to the nucleus, where it dimerizes with the AhR nuclear translocator (ARNT*Arnt*) and binds to xenobiotic response elements in the DNA, initiating hte transcription of target genes^31,32^. The activation of AhR by environmental toxins can lead to the production of ROS, either directly or through the induction of enzymes (for example, CYPs), which metabolize xenobiotics and generate ROS as byproducts^33^. The suppression of AhR in the fructose group could be a negative feedback loop of AhR; fructose-induced ROS may create feedback mechanisms that inhibit AhR signaling. For instance, oxidative stress-induced transcription factors could upregulate inhibitors of AhR (for example, *Ncor*), as evidenced in our report^12^.

SIRT3 activates several mitochondrial enzymes (for example, superoxide dismutase 2 and IDH2)^34^. By deacetylating and activating these enzymes, SIRT3 enhances the ability of cells to neutralize ROS, thereby reducing oxidative stress^35^. In SIRT3-deficient cells or organisms, the lack of deacetylation leads to impaired IDH2 activity, which in turn decreases NADPH being necessary for the reduction of GSH^26,36^. This not only results in an increase in ROS levels but also suppresses the efficiency of detoxification of xenobiotics via GSH, which contributes to various pathologies, including aging-related diseases^37^ and cancers^38^. Based on the findings, it was reasonable to hypothesize that fructose-induced suppression of the SIRT3–IDH2 axis could have affected the enrichment of the chemical carcinogenesis KEGG term predicted in gene ontology analysis (Supplementary Fig. 1).

DNA adduct formation and improper repair can lead to mutations that drive the development of cancer^39^, and defects in DNA repair pathways, such as mutations in BRCA1/2, are associated with increased cancer risk^40^. Moreover, GSH plays a protective role against DNA adduct formation by maintaining the redox environment necessary for proper DNA repair^41^. It is closely linked to both the prevention of DNA damage and the modulation of DNA repair mechanisms, making it a pivotal molecule in the context of genomic stability and cancer biology. In thar regard, the marked increase in γH2AX signifies substantial DNA damage, particularly in the form of double-strand breaks^42^. This is consistent with the elevated protein expression of p53 and cCASP-3 (Fig. 4b), suggesting that the tissue underwent severe DNA damage, triggering p53-mediated apoptotic pathways. The reduction in pATR levels indicates that the cells may no longer be prioritizing DNA repair through the ataxia telangiectasia and Rad3-related (ATR)-mediated pathways^43^ and instead have shifted toward apoptosis (Fig. 4b). As apoptosis progresses, the decrease in cPARP, despite being a marker of late-stage apoptosis, still coincides with the ongoing presence of γH2AX, suggesting a continued DNA fragmentation, a hallmark of the final stages of apoptosis.

The formation of additional colonic ILFs in IDH2-KO mice indicate an enhanced chronic inflammation, as they have been shown to expand in number and size during inflammatory conditions. The ILFs are known to contribute to tumorigenesis by providing a microenvironment conducive to cancer progression^44^. Along with this, the observed increase in γH2AX and PCNA levels in IDH2-KO mice also suggests that these ILFs might be responding to persistent DNA damage and cellular stress. This is consistent with the notion that chronic inflammation in the IDH2-KO mice, combined with ongoing DNA damage (due to PhIP exposure), can promote colonic carcinogenesis^45^.

Overall, as anticipated, our results show that IDH2-KO induces mitochondrial dysfunction, primarily by disrupting the redox balance, a well-documented outcome that we also observed in the present study^46^. Although it was unclear whether PhIP would produce similar effects, our findings demonstrate that it does, probably through the inhibition of pathways such as electron transport, ATP synthesis and heat production by uncoupling proteins and oxidative phosphorylation. Mitochondrial dysfunction often leads to the overproduction of ROS, which can cause DNA damage, including double-strand breaks^47^. This, in turn, triggers the phosphorylation of H2AX, forming γH2AX and initiating the DNA damage response^48^.

Mitochondrial function is essential for maintaining cellular energy homeostasis, and widespread DNA damage, combined with inefficient repair mechanisms, can further impair mitochondrial function. This creates a vicious cycle in which damage to the DNA exacerbates the mitochondrial dysfunction, and the mitochondrial dysfunction increases the DNA damage^49,50^. Based on our comparative analysis, the observed mitochondrial dysfunction is probably the result of a combined effect of IDH2-KO and PhIP treatment. First, IDH2 deficiency leads to an overproduction of ROS, causing DNA damage. Second, PhIP exposure exacerbates this by inducing additional DNA damage, which contributes to further mitochondrial dysfunction. As we demonstrated, fructose suppressed IDH2 activity via SIRT3; this inhibition can lead to worsening oxidative stress and mitochondrial dysfunction. In our study, this notion was strengthened by the combination of IDH2 deficiency and PhIP exposure creating a compounded detrimental effect on mitochondrial function and DNA repair mechanisms, ultimately exacerbating the cellular damages.

The IPA analysis showed several interesting upstream regulators that also account for the phenotypes and potential biofunctions. A total of 166 upstream regulators were predicted activated or inactivated in the IDH2-KO + PhIP group compared with the WT + PhIP group. We focused on the top ten predicted activated and the other ten predicted inhibited upstream regulators (Table 1). The top ten predicted activated upstream regulators and their downstream targets were associated with several biological functions in organismal injury and abnormalities (that is, tumorigenesis of tissue, tumorigenesis of lymphocytes, tumorigenesis of epithelial neoplasm, non-central nervous system malignant neoplasm and nonhematologic malignant neoplasm; Fig. 5c). In addition, the top ten predicted inhibited upstream regulator and their downstream targets were also associated with organismal injury and abnormalities (that is, carcinoma, intra-abdominal organ tumor and non-central nervous system malignant neoplasm; Fig. 5d). These molecules can be categorized into immune and inflammatory responses, cell proliferation, tumor suppression and growth regulation, energy homeostasis and RNA processing and gene regulation. Collectively, upstream analysis suggested that inflammatory and immune, cell proliferation and survival pathways were activated, probably owing to IDH2 deletion and/or PhIP treatment. Simultaneously, the inhibition of tumor suppressors, metabolic regulators and stress responses were inhibited. Such a profile could be seen in cancer, where cells hijack normal proliferative and survival pathways while suppressing controls that would otherwise limit growth^51^. The strong inflammatory signals could also point to a context where the immune system is heavily engaged, potentially working to combat an infection or responding to the tumor microenvironment^52^.

**Table 1:** Top ten upstream regulators predicted activated or inhibited by IDH2 deletion.

Molecules	*Z*-score	*P* value	Target molecules in DEGs
Predicted activated (WT + PhIP versus IDH2-KO + PhIP)			
RICTOR	5.578	1.93 × 10^−18^	ATP6V1G1, CD69, COX4I1, COX5A, COX6A1, Cox6c, COX7B, IFI16, IL1B, NDUFA5, NDUFB10, NDUFB2, NDUFB4, NDUFB5, NDUFB6, NDUFB9, NDUFC1, NDUFC2, Ndufs5, NDUFS6, NDUFV2, OAS2, PPA2, Rpl29 (includes others), RPL30, RPL41, RPLP2, RPS13, RPS24, Uba52, UQCR10, UQCR11, UQCRB, UQCRHL, UQCRQ
CPT1B	4.081	5.4 × 10^−7^	COX4I1, COX5A, Cox6c, COX7B, NDUFA5, NDUFB10, NDUFB2, NDUFB6, NDUFB9, NDUFC1, NDUFS6, NDUFV2, UQCR10, UQCR11, UQCRB, UQCRHL, UQCRQ
TNF	3.948	0.000101	ABCC2, ACE, ADAM8, AGER, ANPEP, APOA1, APOBEC3B, AQP3, BBOX1, BCL2A1, BIRC3, C1QTNF4, CBR3, CCR6, CD69, CDX1, CNR2, COX4I1, CXCR4, CXCR5, CYP1A1, DIO1, DUSP2, ECH1, EGR2, FCER2, FGFRL1, FOSB, FPR1, FTH1, GBP4, GPR18, GSTP1, IDH2, IFI16, IL10RA, IL1B, IL1RL1, IL21R, IL4I1, INHBA, IRF5, KCNQ1OT1, LCP2, LY6D, LYZ, MEFV, MMP10, MMP12, MPC1, MT-CO3, MTTP, NLRP3, OAS2, PARP14, PTPRC, RGCC, RPS13, Slc5a4b, SOD2, ST8SIA4, STAT2, TLR9, TRPC6, TXN2, XDH
IFNG	3.91	2.9 × 10^−8^	ACE, AGER, AGPAT1, AIF1, BACH2, BATF, BCL2A1, BIRC3, C1QA, C1QB, CCR6, CD72, CDX1, CORO1A, COX4I1, CXCR4, DIABLO, DIO1, EGR2, FCER2, FCGR3A/FCGR3B, FGFRL1, FOSB, FTH1, GBP4, GLA, GPT, GSTP1, HAAO, HLA-DOB, IFI16, IFI44, Ighg2b, Ighg3, IGHM, IL10RA, IL1B, IL1RL1, IL4I1, INHBA, IRF5, KCNQ1OT1, LCP2, MEFV, MMP10, MMP12, NAPSA, NLRP3, OAS2, PARP14, PLA2G7, RGCC, SBNO2, Serpina3g (includes others), SIGLEC10, SLC28A2, SLFN12L, SOD2, SP110, SPRR1A, STAT2, TLR9, TMEM171
SPI1	3.506	3.35 × 10^−7^	BTK, CCR6, CD19, CD72, CD79A, CD79B, COX4I1, CSF3R, CTSE, EGR2, FTH1, IFI44, IL1B, LILRB3, LYZ, MME, MS4A1, NDUFB11, PRDX5, PTPRC, SLC24A1, SP110
CLCF1	3.45	1.86 × 10^−9^	CHCHD10, COX4I1, COX5A, MT-ATP6, MT-CO3, MT-ND3, NDUFB11, NDUFB9, NDUFC1, NDUFS6, TIMM10, UQCRQ
NFKB1	3.443	0.00148	BATF, BCL2A1, BIRC3, BLK, CR2, CXCR5, FOSB, IFI16, IGHM, IL10RA, IL1B, MMP10, POU2F2, SOD2, TLR9
ZHX2	3.44	1.04 × 10^−9^	COX5A, COX7B, NDUFAF5, NDUFB2, NDUFB6, NDUFB9, NDUFC1, NDUFS6, UQCR10, UQCR11, UQCRB, UQCRHL
IL33	3.395	2.36 × 10^−10^	ARHGAP30, ATXN1, BATF, BCL2A1, BIRC3, CD69, CD72, CLEC4A, CSF3R, DUSP2, EGR2, FAM162A, FGR, FPR1, IGHM, IKZF3, IL10RA, IL1B, IL1RL1, IL2RG, IL4I1, IRF5, MMP10, MMP12, NLRP3, NT5E, POU2F2, PTPRC, SOD2, UQCC2, UQCRQ
KDM5A	3.357	3.97 × 10^−6^	COX4I1, COX6A1, DUSP2, EXOSC4, GADD45GIP1, MCAT, MRPL11, MRPL12, MRPL17, MRPL55, NDUFA5, NDUFB10, SOD2, TXN2, UQCRQ
Predicted inhibited (WT + PhIP versus IDH2-KO + PhIP)			
TEAD1	−4.796	1.17 × 10^−13^	BBOX1, COX4I1, COX5A, Cox6c, COX7B, ECH1, ETFB, NDUFA12, NDUFA5, NDUFB10, NDUFB2, NDUFB4, NDUFB5, NDUFB6, NDUFC1, NDUFC2, Ndufs5, NDUFS6, NDUFV2, UQCR11, UQCRB, UQCRHL, UQCRQ
DDX5	−3.845	7.07 × 10^−9^	COX5A, COX7B, CXCR4, IL1B, NDUFA12, NDUFA5, NDUFB2, NDUFB4, NDUFB5, NDUFB6, NDUFS6, NFAT5, UQCR10, UQCRB, UQCRQ
SIRT1	−3.825	3.57 × 10^−5^	ABCC2, BIRC3, CD72, CD79B, CORO1A, COX4I1, CYP1A1, IDH2, IFI44, IGHM, Iglc3, IL1B, IL2RG, LRCH1, NDUFA5, NT5E, OAS2, PARP14, RNF213, SOD2, SP110, TBC1D10C, Trim30a/Trim30d
CAB39L	−3.742	2.82 × 10^−15^	COX4I1, NDUFA12, NDUFB10, NDUFB11, NDUFB2, NDUFB5, NDUFB9, NDUFC1, NDUFS6, NDUFV2, UQCR10, UQCR11, UQCRB, UQCRQ
PPARGC1A	−3.619	1.05 × 10^−5^	AGER, COX4I1, COX5A, COX7B, CYP1A1, DIO1, IL1B, INHBA, NCEH1, NDUFB4, NDUFB5, NDUFV2, NLRP3, PGAM1, PLA2G7, PRDX5, RECQL5, S100A1, SLC24A1, SLC25A39, SMC5, SOD2, TLR9, TXN2, XDH
IRF2BP2	−3.582	1.16 × 10^−6^	APOA1, BCL2A1, CD72, CLEC6A, FCGR3A/FCGR3B, IFI16, Ighg3, IGHM, IL2RG, LILRB3, LY6D, STAP1, Trim30a/Trim30d
IGBP1	−3.573	1.05 × 10^−11^	COX6A1, Cox6c, COX7B, NDUFA12, NDUFB10, NDUFB11, NDUFB2, NDUFB5, NDUFB6, NDUFB9, Ndufs5, UQCR11, UQCRQ
CITED2	−3.208	9.02 × 10^−5^	CD69, FCGR3A/FCGR3B, GBP4, IFI16, IFI44, IL1B, LCP2, LRCH1, PARP14, Serpina3g (includes others), SLC28A2, SLFN12L, TMEM171, Trim30a/Trim30d
TFRC	−3.207	3.73 × 10^−7^	FTH1, MMP12, NDUFB10, NDUFB2, NDUFB4, NDUFB5, NDUFB6, NDUFB9, Ndufs5, NDUFS6, SPRR1A, UQCR11, UQCRHL, UQCRQ
GFER	−3.162	3.66 × 10^−10^	CHCHD10, COX4I1, COX5A, COX6A1, NDUFB9, NDUFV2, UQCR10, UQCRB, UQCRHL, UQCRQ

Abbreviations: DEG, differentially expressed genes; IDH2, isocitrate dehydrogenase 2; KO, knockout; PhIP, 2-amino-1-methyl-6-phenylimidazo(4,5-b)pyridine; WT, wild type

We investigated the molecular mechanisms by which fructose contributes to PhIP-induced colon carcinogenesis, with a specific emphasis on the SIRT3–IDH2 axis. Our results collectively show that fructose disrupts mitochondrial function and redox homeostasis by suppressing SIRT3, which normally deacetylates and activates IDH2. This suppression of SIRT3 leads to decreased NADPH production, weakening antioxidant defenses and increasing oxidative stress. Moreover, our experiments with IDH2-KO mice reveal that these mice exhibit heightened vulnerability to DNA damage and tumorigenesis when exposed to the PhIP, both in short- and long-term exposure models. These findings highlight the critical role of the SIRT3–IDH2 axis in preserving mitochondrial function and detoxification capacity, both of which are impaired by fructose consumption. The combined effects of fructose-induced oxidative stress and reduced detoxification probably play a central role in promoting colon carcinogenesis, especially in the presence of carcinogens such as PhIP.

In addition to implications of fructose on SIRT3–IDH2 axis, emerging evidence suggests that mitochondrial redox imbalance is not merely a downstream consequence but may also function as an initiating factor contributing to genomic instability and tumor development^53^. Our results extend this concept by showing that the suppression of SIRT3 and IDH2 could be triggered not only by excessive fructose intake but also by other metabolic stressors such as high-fat diet and obesity-associated inflammation, which have been shown to impair SIRT3-mediated antioxidant defense mechanisms^20^. These insights suggest that the SIRT3–IDH2 axis represents a common vulnerability point in metabolically stressed tissues, particularly the colon, and provide a broader perspective on how targeting mitochondrial integrity could offer new avenues for colon cancer prevention. Overall, these findings suggest that fructose-induced mitochondrial dysfunction through the SIRT3–IDH2 axis may critically contribute to carcinogenesis, particularly under conditions of additional dietary carcinogen exposure. This study underscores the importance of further investigating how fructose modulates these pathways and highlights potential therapeutic targets to reduce the risks associated with high fructose intake, especially in relation to colon cancer.

It is important to acknowledge that previous studies have reported the carcinogenic effects of IDH2, which seem contradictory to our findings. However, those studies primarily focused on xenograft or IDH2-mutation models, rather than examining the role of IDH2 in the initiation stage of cancer^54–56^. By contrast, our study provides evidence that IDH2 deficiency may promote cancer initiation from a different perspective. In carcinogen-free IDH2-KO mice, we observed a significant upregulation of genes involved in DNA damage and repair signaling in colon tissue. Furthermore, multiomics datasets mutually indicated enhanced carcinogenesis-associated signaling pathways and upstream molecules in these mice. Collectively, our findings suggest that IDH2 may play a protective role during the early stages of cancer development, offering a fresh perspective for cancer research.

One of our major challenges was that the effects of fructose on PhIP-induced colon carcinogenesis were not directly examined. Despite the lack of a specific experiment, multiple previous reports using different models strongly suggest that fructose amplifies carcinogenesis^5,57^. In addition, a limitation of this study is the absence of functional validation of mitochondrial activity (for example, ATP production) and the lack of direct assessment of fructose’s effects on the AhR signaling pathway and the SIRT3–IDH2 axis in colon tissues. These analyses were not feasible owing to the limited availability of biological samples, as the collected tissues were comprehensively used for detailed histopathological and molecular evaluations. Moreover, we focused on the liver because the biotransformation and excretion of PhIP primarily occur in hepatic tissue, which is central to its metabolic activation and detoxification. Nevertheless, our study also presents important benefits, as multiple omics datasets from three sets of animal experiments including KO mice model were comprehensively analyzed and validated, which provides valuable evidence for different perspectives on WT IDH2 in the initiation of cancer, and a strong foundation for future research in various fields such as cancer research.

In summary, our study highlights the critical role of the SIRT3–IDH2 axis in the context of chemical-induced colon carcinogenesis. Using unbiased multiomics approaches as well as unique models (for example, SIRT3 and IDH2-KO models), we demonstrated that fructose suppresses SIRT3 expression and IDH2 activity, leading to impaired mitochondrial detoxification, increased oxidative stress and a weakened DNA damage response. These effects, in turn, exacerbate DNA damage and promote tumorigenesis, particularly in the absence of IDH2. This study therefore provides a strong foundation for targeting this pathway as a preventive strategy to mitigate the carcinogenic effects of dietary fructose and related environmental factors.

## Supplementary information

Supplementary Information
