# Supplementary material for: SIRT3–IDH2 axis is a target of dietary fructose: implication of IDH2 as a key player in dietary carcinogen toxicity in mice colon
Source: Exp Mol Med. 2025 Nov 13;57(11):2643–56. doi: 10.1038/s12276-025-01584-0 (PMC12686511; doi:10.1038/s12276-025-01584-0)
Supplement: Supplementary file 1 — Supplementary Information [file 12276_2025_1584_MOESM1_ESM.pdf]

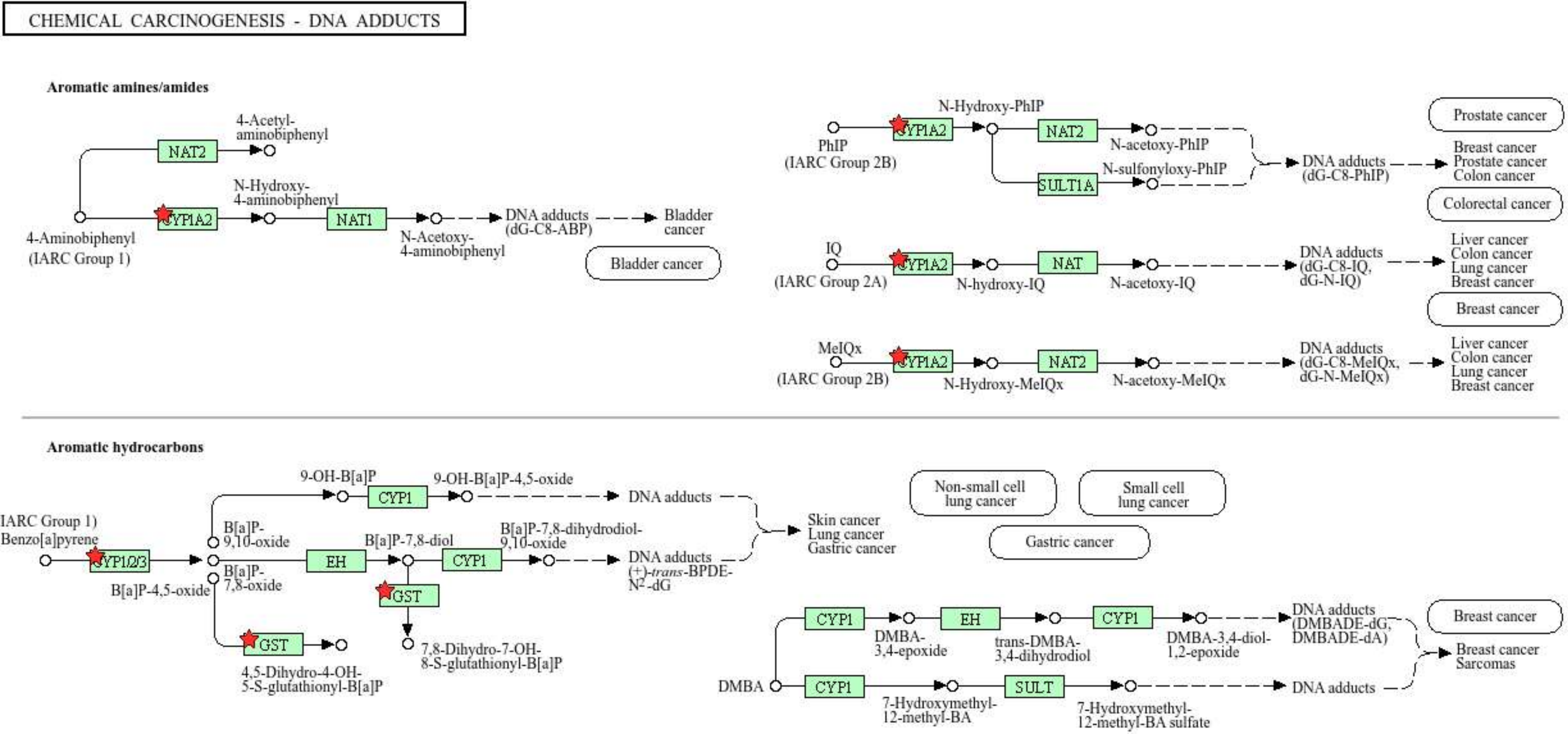

Supplementary Figure 1. Kyoto Encyclopedia of Genes and Genomes (KEGG) pathway: Chemical Carcinogenesis – DNA adducts.
A KEGG pathway 'Chemical Carcinogenesis – DNA adduct' enriched liver tissues from mice fed fructose water, which was retrieved from transcriptome for KEGG pathway terms.
Abbreviations: KEGG, Kyoto Encyclopedia of Genes and Genomes

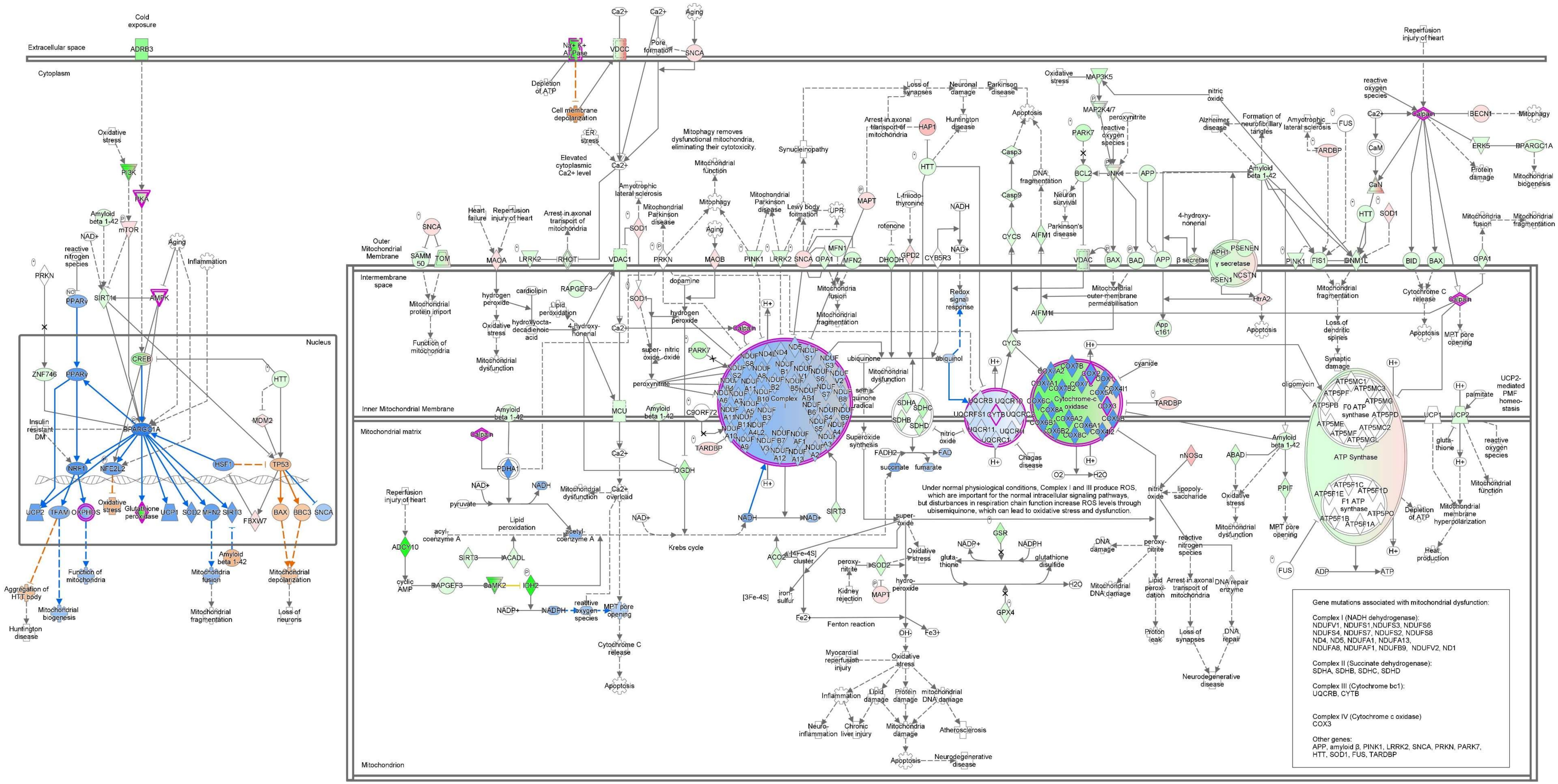

Supplementary Figure 2. Canonical Pathway: Mitochondrial dysfunction.

Colonic transcriptomics reveals that isocitrate dehydrogenase 2 (IDH2) knockout (KO)-mediated mitochondrial dysfunction is likely related to the suppression of Complex IV and V compared to wild type (WT) mice. In IDH2 KO mice, transcriptomic analysis revealed marked inhibition of genes involved in electron transport chain Complex IV (e.g., *Cox5b*, *Cox6a2*) and ATP synthase complex (e.g., *Atp5o*), as indicated by downregulation of those targets. Inhibition of *Ndufa2* and *Ndufb8* also suggests compromised NADH dehydrogenase function, collectively pointing to impaired mitochondrial respiration and energy production. Green and red colors indicate experimentally observed inhibition or activation of genes or group of genes, respectively. Blue and orange colors are predicted inhibited or activated genes or group of genes, respectively.

Abbreviations: IDH2, isocitrate dehydrogenase 2; KO, knockout; WT, wild type

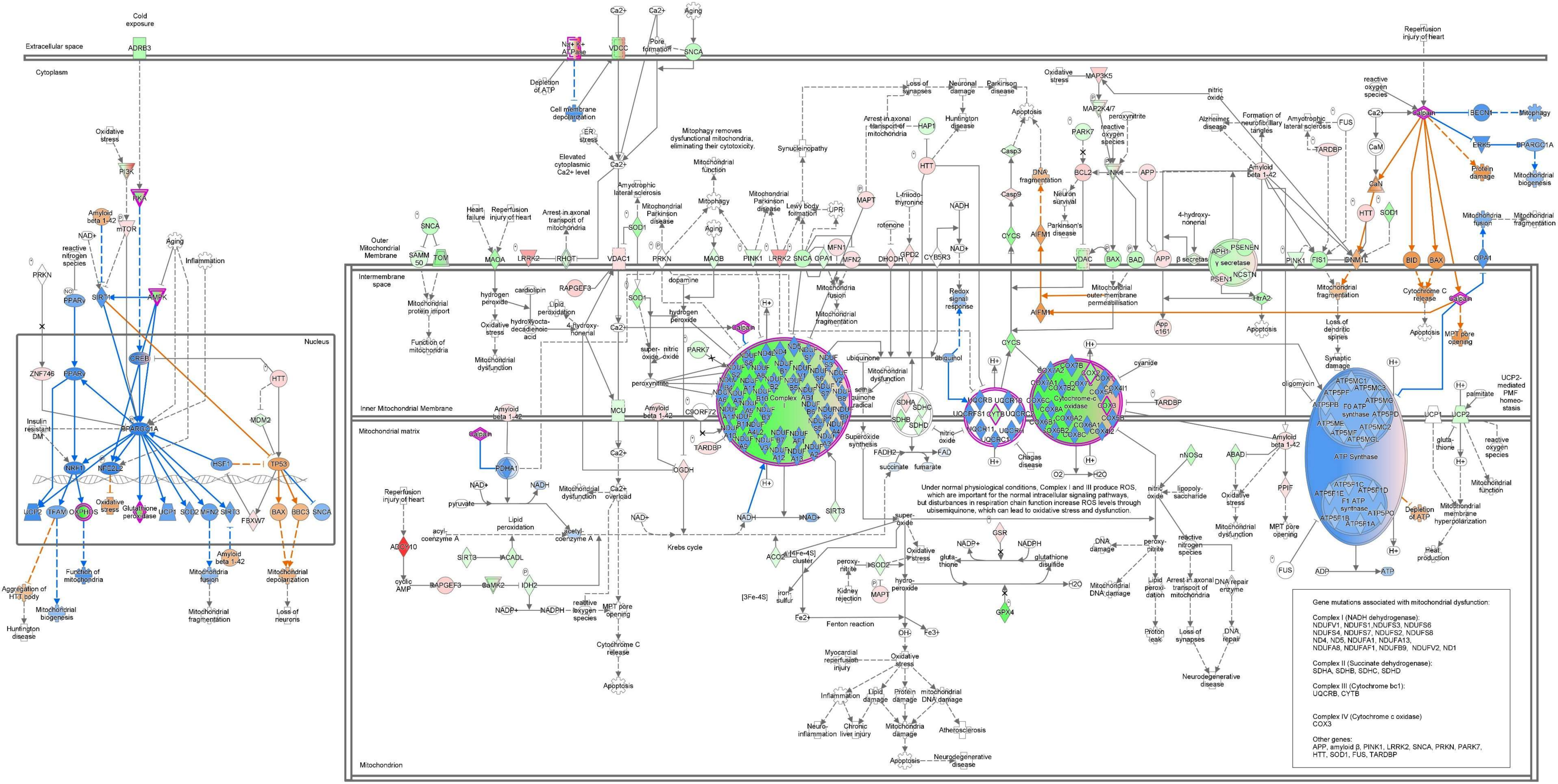

Supplementary Figure 3. Canonical Pathway: Mitochondrial dysfunction.

Colonic transcriptomics reveals that 2-amino-1-methyl-6-phenylimidazo(4,5-b)pyridine (PhIP)-mediated mitochondrial dysfunction is likely related to the suppression of Complex I and IV in wilt type (WT) mice. Exposure to PhIP led to suppression of *Ndufb8*, *Ndufa10*, and *Cox6a1*, which are essential components of Complex I and IV, respectively. These changes imply diminished electron transfer capacity and elevated mitochondrial ROS production. Upregulation of apoptotic genes such as *Bax* and activation of cytochrome c-mediated signaling also indicate enhanced mitochondrial damage response. Green and red colors indicate experimentally observed inhibition or activation of genes or group of genes, respectively. Blue and orange colors are predicted inhibited or activated genes or group of genes, respectively. Abbreviations: IDH2, isocitrate dehydrogenase 2; KO, knockout; PhIP, 2-amino-1-methyl-6-phenylimidazo(4,5-b)pyridine; WT, wild type

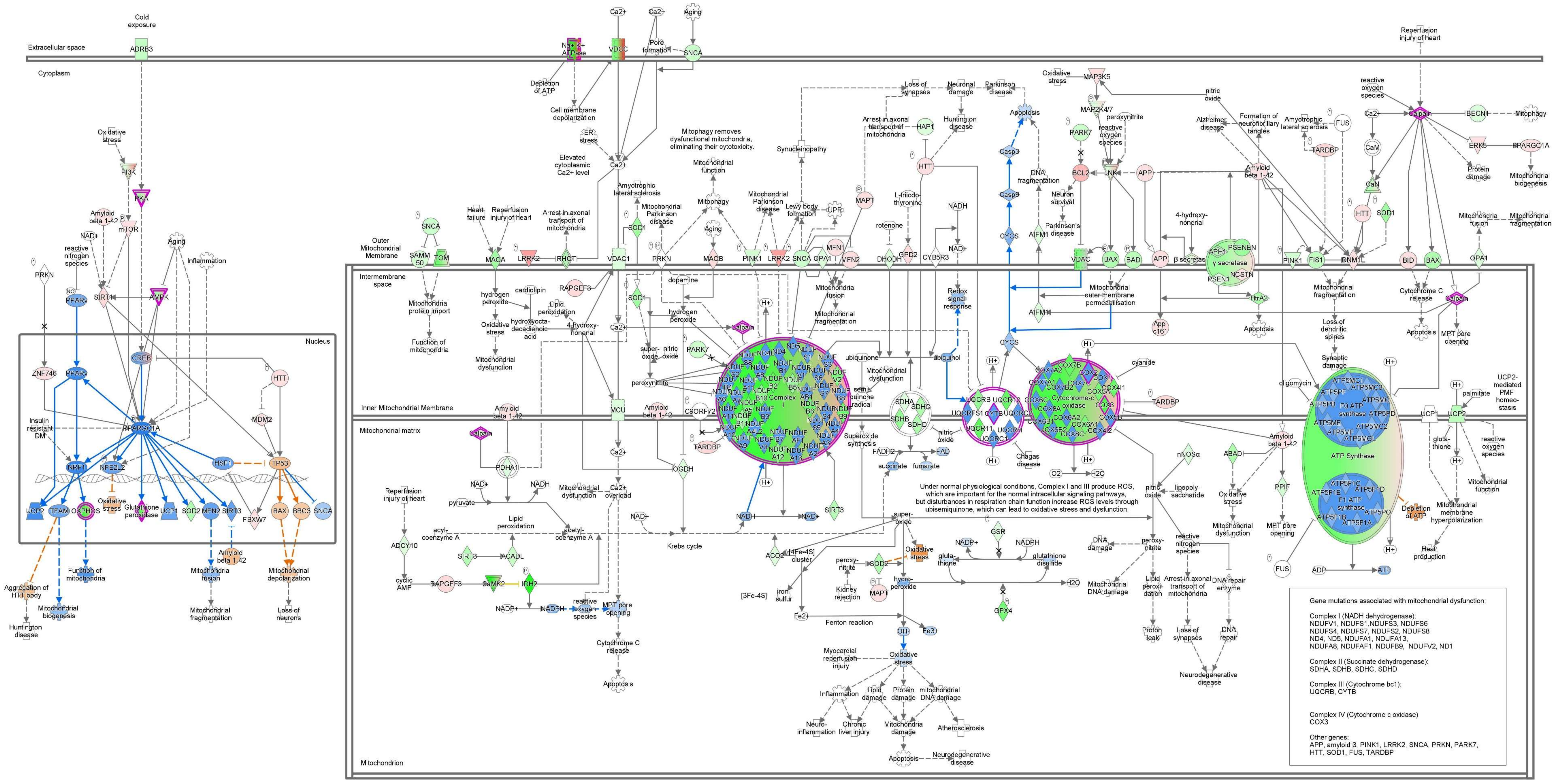

Supplementary Figure 4. Canonical Pathway: Mitochondrial dysfunction.

Colonic transcriptomics reveals that isocitrate dehydrogenase 2 (IDH2) knockout (KO) exacerbates 2-amino-1-methyl-6-phenylimidazo(4,5-b)pyridine (PhIP)-mediated mitochondrial dysfunction via overall suppression of the electron transport chain. Combined IDH2 KO and PhIP exposure resulted in pronounced inhibition of multiple complexes in the electron transport chain, including *Ndufs3*, *Cox7a2*, *Uqcrc1*, and *Atp5po*, suggesting synergistic mitochondrial suppression. Increased inhibition of *Sdha* and downregulation of antioxidant enzyme *Gpx4* indicate impaired oxidative phosphorylation and redox imbalance, consistent with the severe mitochondrial dysfunction phenotype observed in vivo. Green and red colors indicate experimentally observed inhibition or activation of genes or group of genes, respectively. Blue and orange colors are predicted inhibited or activated genes or group of genes, respectively.

Abbreviations: IDH2, isocitrate dehydrogenase 2; KO, knockout; PhIP, 2-amino-1-methyl-6-phenylimidazo(4,5-b)pyridine

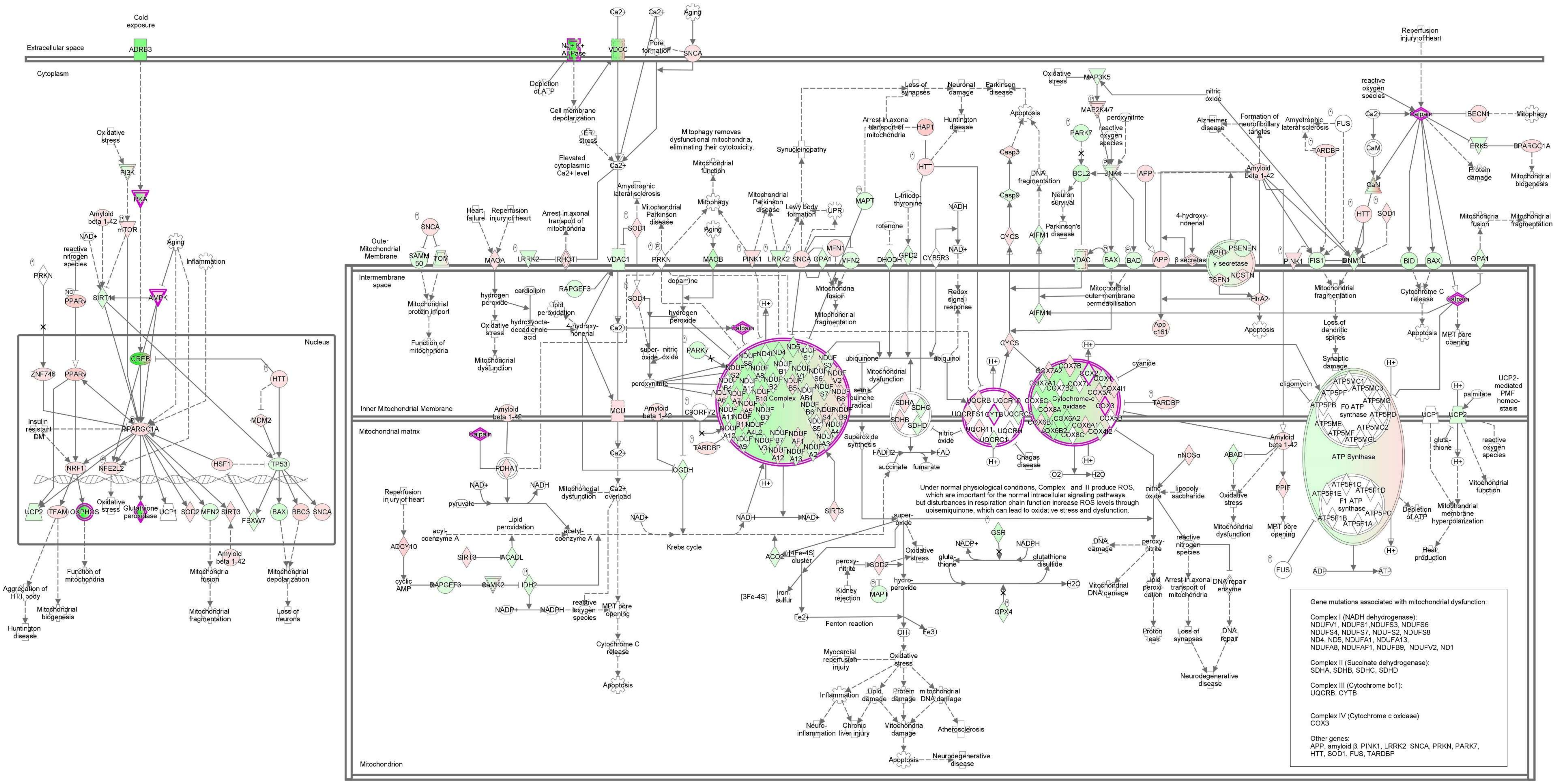

Supplementary Figure 5. Canonical Pathway: Mitochondrial dysfunction.
Colonic transcriptomics reveals that 2-amino-1-methyl-6-phenylimidazo(4,5-b)pyridine (PhIP) treatment to the isocitrate dehydrogenase 2 (IDH2) knockout (KO) mice did not significantly alter mitochondrial dysfunction in colon compared to IDH2 KO mice. Transcriptomic differences between PhIP-treated IDH2 KO and untreated IDH2 KO mice were relatively minor. Only subtle changes were observed in a few components such as *Cox5a* and *Ndufa5*, suggesting that the mitochondrial dysfunction induced by IDH2 deficiency may already be maximized, with PhIP having limited additive effect in this context. Green and red colors indicate experimentally observed inhibition or activation of genes or group of genes, respectively. Blue and orange colors are predicted inhibited or activated genes or group of genes, respectively. Abbreviations: IDH2, isocitrate dehydrogenase 2; KO, knockout; PhIP, 2-amino-1-methyl-6-phenylimidazo(4,5-b)pyridine

Supplementary Table 1. Activated or inhibited canonical pathways1 in colon tissues by IDH2 knockout

Canonical Pathways	-log(p-value)	z-score[2]
Interferon alpha/beta signaling	10.7	3.606
Role of Hypercytokinemia/hyperchemokinemia in the Pathogenesis of Influenza	8.53	2.887
ISGylation Signaling Pathway	5.52	2.53
Pathogen Induced Cytokine Storm Signaling Pathway	2.61	2.496
DDX58/IFIH1-mediated induction of interferon-alpha/beta	2.29	2.236
Activation of IRF by Cytosolic Pattern Recognition Receptors	5.45	2.121
Neuroinflammation Signaling Pathway	3.76	2.111
Interferon Signaling	3.85	2
Antigen Presentation Pathway	2.67	2
SPINK1 Pancreatic Cancer Pathway	2	2
Immunogenic Cell Death Signaling Pathway	1.42	2
Death Receptor Signaling	1.33	2
Glucose metabolism	1.58	-2
Coronavirus Pathogenesis Pathway	2.07	-2.121
The citric acid (TCA) cycle and respiratory electron transport	2.98	-2.236
O-linked glycosylation	1.69	-2.236
Ion channel transport	2.93	-2.333
Gap Junction Signaling	1.39	-2.333
Serotonin Receptor Signaling	2.55	-2.84

[1]Activity states were predicted by the IPA software using differentially expressed genes.

[2]Calculated activity score. Positive and negative values indicate predicted activated and predicted inhibited, respectively.
